# A phosphoinositide map at the shoot apical meristem in *Arabidopsis thaliana*

**DOI:** 10.1186/s12915-018-0490-y

**Published:** 2018-02-07

**Authors:** Thomas Stanislas, Matthieu Pierre Platre, Mengying Liu, Léa E. S. Rambaud-Lavigne, Yvon Jaillais, Olivier Hamant

**Affiliations:** Laboratoire de Reproduction et Développement des Plantes, Université de Lyon, ENS de Lyon, UCBL, INRA, CNRS, 46 Allée d’Italie, 69364 Lyon, Cedex 07 France

**Keywords:** Shoot apical meristem, Phosphatidylinositol phosphate, Organogenesis, Stem cell, Morphogenesis, Mechanotransduction, Arabidopsis

## Abstract

**Background:**

In plants, the shoot apical meristem (SAM) has two main functions, involving the production of all aerial organs on the one hand and self-maintenance on the other, allowing the production of organs during the entire post-embryonic life of the plant. Transcription factors, microRNA, hormones, peptides and forces have been involved in meristem function. Whereas phosphatidylinositol phosphates (PIPs) have been involved in almost all biological functions, including stem cell maintenance and organogenesis in animals, the processes in meristem biology to which PIPs contribute still need to be delineated.

**Results:**

Using biosensors for PI4P and PI(4,5)P_2_, the two most abundant PIPs at the plasma membrane, we reveal that meristem functions are associated with a stereotypical PIP tissue-scale pattern, with PI(4,5)P_2_ always displaying a more clear-cut pattern than PI4P. Using *clavata3* and *pin-formed1* mutants, we show that stem cell maintenance is associated with reduced levels of PIPs. In contrast, high PIP levels are signatures for organ-meristem boundaries. Interestingly, this pattern echoes that of cortical microtubules and stress anisotropy at the meristem. Using ablations and pharmacological approaches, we further show that PIP levels can be increased when the tensile stress pattern is altered. Conversely, we find that *katanin* mutant meristems, with increased isotropy of microtubule arrays and slower response to mechanical perturbations, exhibit reduced PIP gradients within the SAM. Comparable PIP pattern defects were observed in phospholipase A3β overexpressor lines, which largely phenocopy *katanin* mutants at the whole plant level.

**Conclusions:**

Using phospholipid biosensors, we identified a stereotypical PIP accumulation pattern in the SAM that negatively correlates with stem cell maintenance and positively correlates with organ-boundary establishment. While other cues are very likely to contribute to the final PIP pattern, we provide evidence that the patterns of PIP, cortical microtubules and mechanical stress are positively correlated, suggesting that the PIP pattern, and its reproducibility, relies at least in part on the mechanical status of the SAM.

**Electronic supplementary material:**

The online version of this article (10.1186/s12915-018-0490-y) contains supplementary material, which is available to authorized users.

## Background

All aerial plant organs are initiated in a group of dividing cells called shoot apical meristem (SAM). In contrast to animals, plants can generate new organs during their entire life because SAMs are self-maintained. In short, SAMs have two main functions, namely organogenesis and self-maintenance.

SAMs display a stereotypical histology with a central zone containing slowly dividing cells, corresponding to the true stem cells, and a peripheral zone, with rapidly dividing cells and a dense cytoplasm, corresponding to the organogenetic ring (e.g., [1, 2]). From the 1980s onwards, the genetic bases of meristem function have been elucidated thanks to mutants displaying defects in stem cell maintenance or organogenetic potential. Typically, increased and reduced maintenance of stem cell pools in *clavata3* (*clv3*) and *wuschel* (*wus*) mutants, respectively, led to the discovery of a feedback loop in which the transcription factor WUS, together with HAM1-2, promotes stem cell-ness as well as its own negative regulator, through the CLV3 ligand and CLV1-CLV2-CRN receptors [3]. In the 1990s, this genetic network was further shown to incorporate hormones, and notably auxin, which is thought to be the main inducer of organ positioning and emergence. More specifically, the *PIN-FORMED 1* gene (*PIN1*) encodes an auxin efflux carrier and extensive literature (e.g., [4–6]) conclusively showed that organogenesis at the SAM requires PIN1-dependent auxin transport towards the site of primordium (i.e., incipient organ) initiation. Other hormones were shown to contribute to organogenesis, albeit to a lower degree (e.g., [7–10]). More recently, the structural elements of meristematic cells were analyzed and shown to consist of a stereotypical tissue-scale pattern of cortical microtubule arrays (and arguably cellulose deposition), with isotropic orientations in the central zone and circumferential orientations in the peripheral zone [11, 12]; patterns of cell wall components and regulators were also identified [13]. Among the structural components of plant cells, the plasma membrane has received very little attention at the shoot meristem. Yet, its position at the interface between the cell wall and the cytoplasm may make its composition a key coordinating feature for the meristem, at the nexus between biochemical and mechanical cues.

In contrast to animal embryos, where boundaries are the main inducers of long-range gradients, acting as master orchestrators of differentiation, the exact contribution of organ boundaries to meristem function is often overlooked [14], and no long-range gradients from boundaries have been revealed. Typically, auxin activity is minimal in boundaries [15], and many boundary-specific genes exhibit a sharp expression pattern [14]. Proteins may diffuse between cells and generate such gradients, like WUS, generating a decreasing gradient from the organizing center of the meristem [16], or cytokinin repressor AHP6, generating a decreasing gradient from emerging organs [7]. However, so far, none of the boundary factors have been shown to exhibit such a long-range diffusion pattern. In fact, presumptive long-range gradients from meristem boundaries might better match biomechanical patterns – mechanical stress is predicted to be high and directional at the boundary (notably because the rapidly growing organ compresses the meristem as tissue folds) and exhibits a long-range decreasing gradient, becoming lower and more isotropic away from the organ boundary. Consistently, cortical microtubule arrays, which align along maximal tensile stress directions, are highly aligned at the boundary and this behavior becomes increasingly noisier from the peripheral zone to the central zone [11]. The promoter activities of *SHOOT MERISTEMLESS* and *CUP-SHAPED COTYLEDON 3* have been associated with such a gradient of mechanical stress [17, 18], but how these long-range mechanical features may interfere with specific meristem regulators or function remains largely unknown.

Phospholipids have been involved in the control of cytoskeleton cortical anchoring and dynamics both in plants and animals (e.g., [19, 20]). Membrane composition, and more specifically phosphatidylinositol phosphates (PIPs), has been associated with mechanosensing in animal cells, notably through their impact on actin dynamics [20], mechanosensors like integrin [21], and mechanosensitive channels [22]. Yet, how PIP accumulation relates to mechanosensing remains an open question in both plants and animals.

In plants, PI3P is enriched in late endosomes, while PI4P and PI(4,5)P_2_ are most abundant at the plasma membrane [23]. Taking advantage of recently characterized PIP biosensors, we reveal here the existence of a stereotypical tissue-scale pattern of plasma membrane-associated PIPs (PI4P and PI(4,5)P_2_), also matching the predicted mechanical stress pattern, at the SAM.

## Results

### A stereotypical PI4P and PI(4,5)P_**2**_ pattern at the SAM

To reveal the pattern of PI4P and PI(4,5)P_2_ at the shoot apex, we generated transgenic lines with a reporter for PI4P (P4M domain from the Legionela protein SiDM: P4M^SiDM^) and PI(4,5)P_2_ (PH domain from Rat Phospholipase C, PLC: PH^PLC^) fused to the yellow fluorescent protein mCitrine [23, 24]. These biosensors were expressed under the control of the *pPDF1* promoter, which restricts expression in the epidermis and is rather homogeneously active across the different SAM regions [17]. To extract signal intensity in each cell, we used the MorphoGraphX software [25] and generated corresponding heatmaps (see Methods). Using this pipeline, we could also obtain heatmaps of Gaussian curvature, based on the membrane-derived meristem topography, allowing us to distinguish four zones in the shoot apex (Additional file 1: Figure S1). More specifically, the boundary domain is easily recognized by its negative Gaussian curvature; the organs encompass all cells from primordia outside of the boundary domain (old organs are excluded); both the central zone and peripheral zone exhibit positive curvature and are delineated by the boundary domain; and the separation between central zone and peripheral zone was determined arbitrarily assuming that the thickness of the ring representing the peripheral zone is roughly equal to the diameter of the central zone (Additional file 1: Figure S1).

**Fig. 1:**
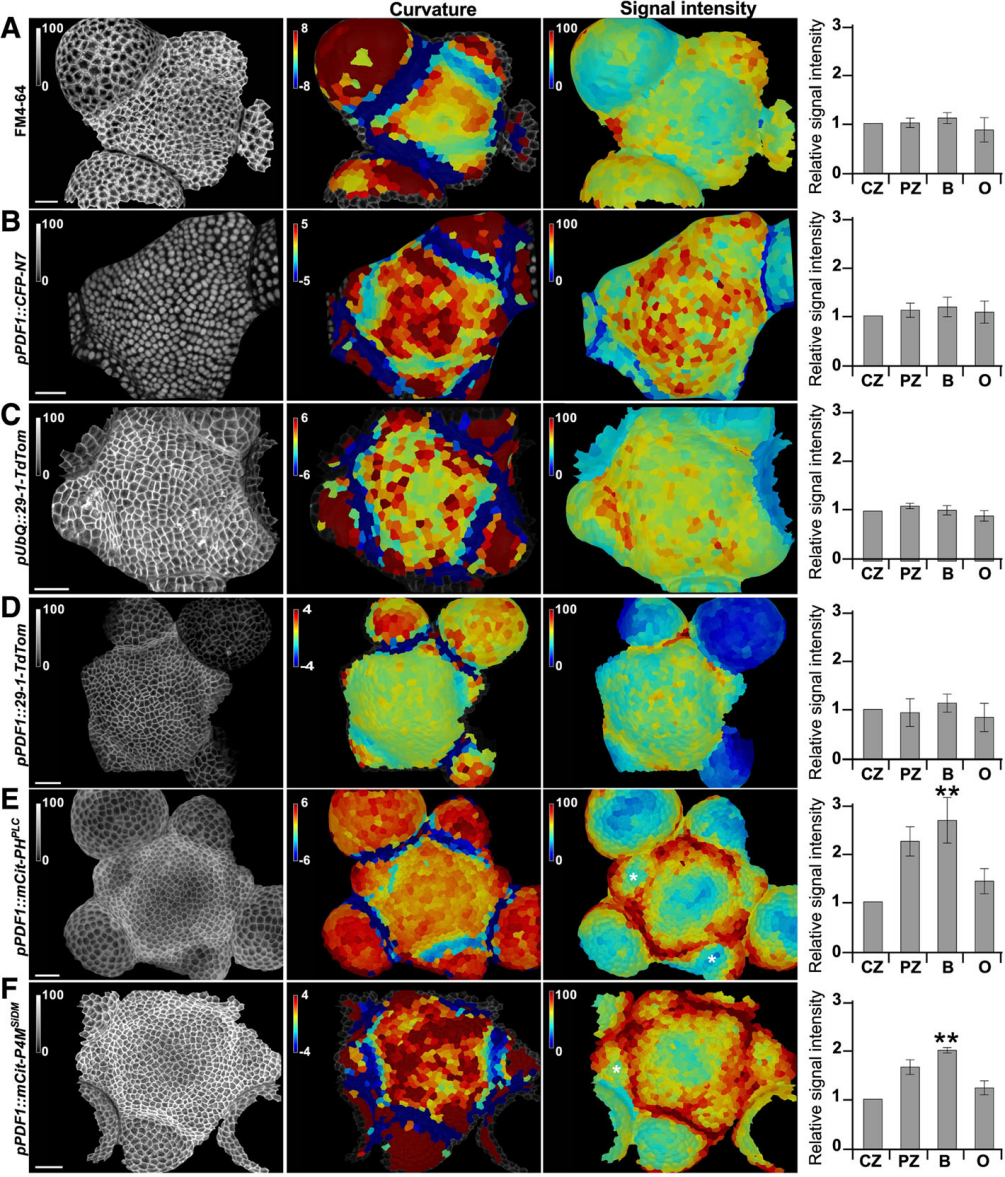
A stereotypical PIP pattern at the shoot apical meristem. Epidermal signal intensity (left panel, %) in shoot meristems (**a**) stained with FM4-64, (**b**) expressing *pPDF1::CFP-N7*, (**c**) expressing *pUbQ::29-1-TdTomato*, (**d**) expressing *pPDF1::29-1-TdTomato*, (**e**) expressing *pPDF1::mCitrine-PH*^*PLC*^, and (**f**) expressing *pPDF1::mCitrine-P4M*^*SiDM*^. Gaussian curvature (central panel, unit: 10^-3^ μm^-2^) and signal intensity (right panel, %) were extracted and signal intensity was quantified in the central zone (CZ), in the peripheral zone (PZ), in the boundary (B), and in young organs (O). Comparisons of signal intensity between the central zone and other domains were performed and statistical significance was tested using a bilateral Student test (**P < 0.001). Scale bar, 20 μm

To check for potential bias in our imaging pipeline or in the tissue itself, we first analyzed markers with predicted homogeneous patterns. We quantified the signal intensity of a lipophilic marker (FM4-64) at the meristem epidermis and could not detect significant differences in signal intensities between the four domains listed above (Fig. 1a, *P* > 0.05). Similarly, the signal intensity of transgenic membrane marker *pUbQ:29-1-TdTomato* (LOW TEMPERATURE INDUCED 6b integral protein with two transmembrane passes [26] under the control of *UBIQUITIN* promoter) was relatively homogeneous between domains at the epidermis (Fig. 1c, *P* > 0.05; Additional file 2: Table S1 and Additional file 3: Table S2). Note that, in both contexts, local heterogeneities in signal intensities could be observed between adjacent cells, and that the variability in intensity was higher in organs and boundaries than in the meristem (central zone and peripheral zone). We also confirmed that the *PDF1* promoter provides a rather homogeneous pattern in the SAM, using a *pPDF1::CFP-N7* line ([17], Fig. 1b, *P* > 0.05; Additional file 2: Table S1 and Additional file 3: Table S2) and a *pPDF1::29-1-TdTomato* line (Fig. 1d, *P* > 0.05; Additional file 2: Table S1 and Additional file 3: Table S2). Note that the *pPDF1* promoter seemed slightly induced in a narrow file of cells at the bottom of old boundaries and was less active in older organs.

Next, to quantify this pattern, we measured the total signal intensity of the PI(4,5)P_2_ biosensor per cell. The ratio of signal intensity between central and peripheral zones was 2.21, and the ratio between the central zone and the boundary was 2.63 (Fig. 1e, n = 4, *P* < 0.001; Additional file 2: Table S1 and Additional file 3: Table S2). We also checked signal intensity for our PI4P biosensor, and found qualitatively similar results, wherein the central zone had the lowest signal intensity, while the boundary and peripheral zone had the highest signal intensity (1.97 and 1.63 times higher than central zone, respectively) (Fig. 1f, n = 4, *P* < 0.001; Additional file 2: Table S1 and Additional file 3: Table S2). Thus, we find that the minima of Gaussian curvature (typically, negative curvature in organ-boundary domain) correlates with the highest PIP levels, whereas the maximal Gaussian curvature (tip of primordia or meristem) does not correlate with high PIP levels (Fig. 1e, f, asterisks). In other words, this suggests that, when a bump appears in the peripheral zone, PIP concentration decreases, whereas PIP accumulates in the boundary domain.

**Fig. 2:**
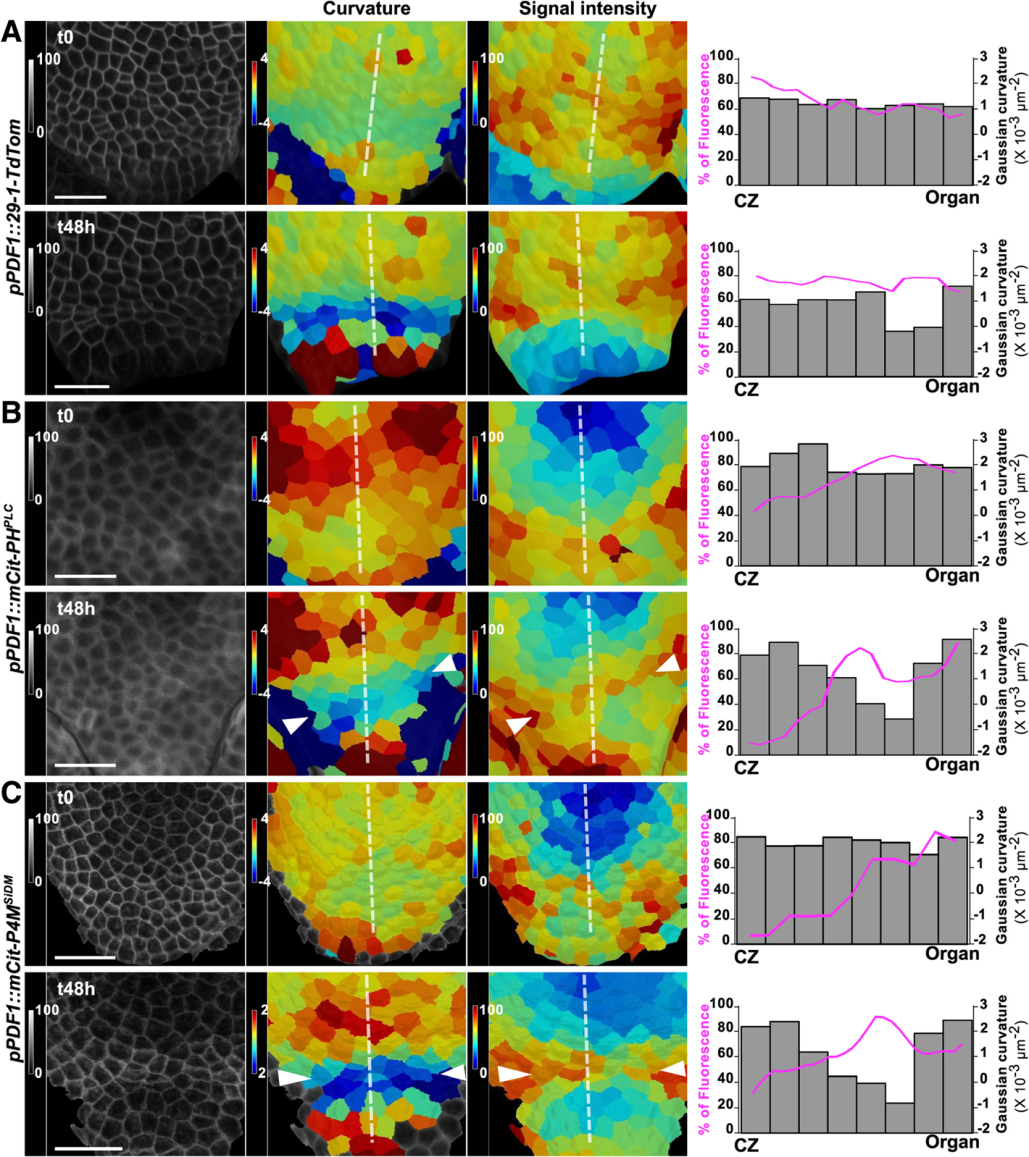
Dynamics of PIP accumulation at the shoot apical meristem. Epidermal signal intensity (left panel, %) in shoot meristems expressing (**a**) *pPDF1::29-1-TdTomato*, (**b**) *pPDF1::mCitrine-PH*^*PLC*^, and (**c**) *pPDF1::mCitrine-P4M*^*SiDM*^. Gaussian curvature (central panel, unit: 10^-3^ μm^-2^) and signal intensity (right panel, %) were extracted and plotted following the dotted white line. Scale bar, 20 μm

To check that scenario, we next analyzed the dynamics of PI4P and PI(4,5)P_2_ biosensor patterns over time. Upon dissection, we maintained stem apices on growth medium supplemented with cytokinin and vitamins (see Methods) allowing us to observe meristem growth and organogenesis over several days [27]. Using this protocol, we revealed a reproducible dynamics of PIP accumulation over time, with a maintenance of low PI4P and PI(4,5)P_2_ at the central zone and an increase of PI4P and PI(4,5)P_2_ in boundaries as organ emerged (Fig. 2; note that the analysis of cropped images increases the relative differences in signal intensities; for a comparison of patterns between lines, see Fig. 1).

Interestingly, within domains, local heterogeneities could be observed. For instance, the shape of the PI4P and PI(4,5)P_2_ depletion zone at the central zone also evolved over time, following the emergence of neighboring organs (Fig. 2). The lateral sides of the boundary domain always displayed a higher signal intensity than the center of the boundary domain (Fig. 2, arrowheads).

Altogether this suggests that PI4P and PI(4,5)P_2_ are associated with meristem function, with low PIP levels in the stem cell maintenance domain (central zone) and high PIP levels in the organogenetic ring (peripheral zone and boundary). Because of the apparent correlation between organogenesis and PIP accumulation, a contribution of auxin in the generation of this pattern may be inferred, notably knowing that the peripheral zone is auxin rich, while the central zone is auxin rich but lacks most of the auxin transduction machinery [15]. Furthermore, PI5P kinase has been shown to be auxin inducible in roots [28] and auxin can induce PI(4,5)P_2_ production in embryos and seedlings [29]. However, the observation that PIP accumulation is not maintained when primordia emerge (in contrast to auxin) and the presence of high PIP levels in boundaries, where auxin levels are low, suggest that the relation between PIP and organogenesis is more complex. To investigate this further, we next affected organogenesis and auxin patterns and observed the consequence on the PIP pattern.

### A PIP pre-pattern at the SAM

**Fig. 3:**
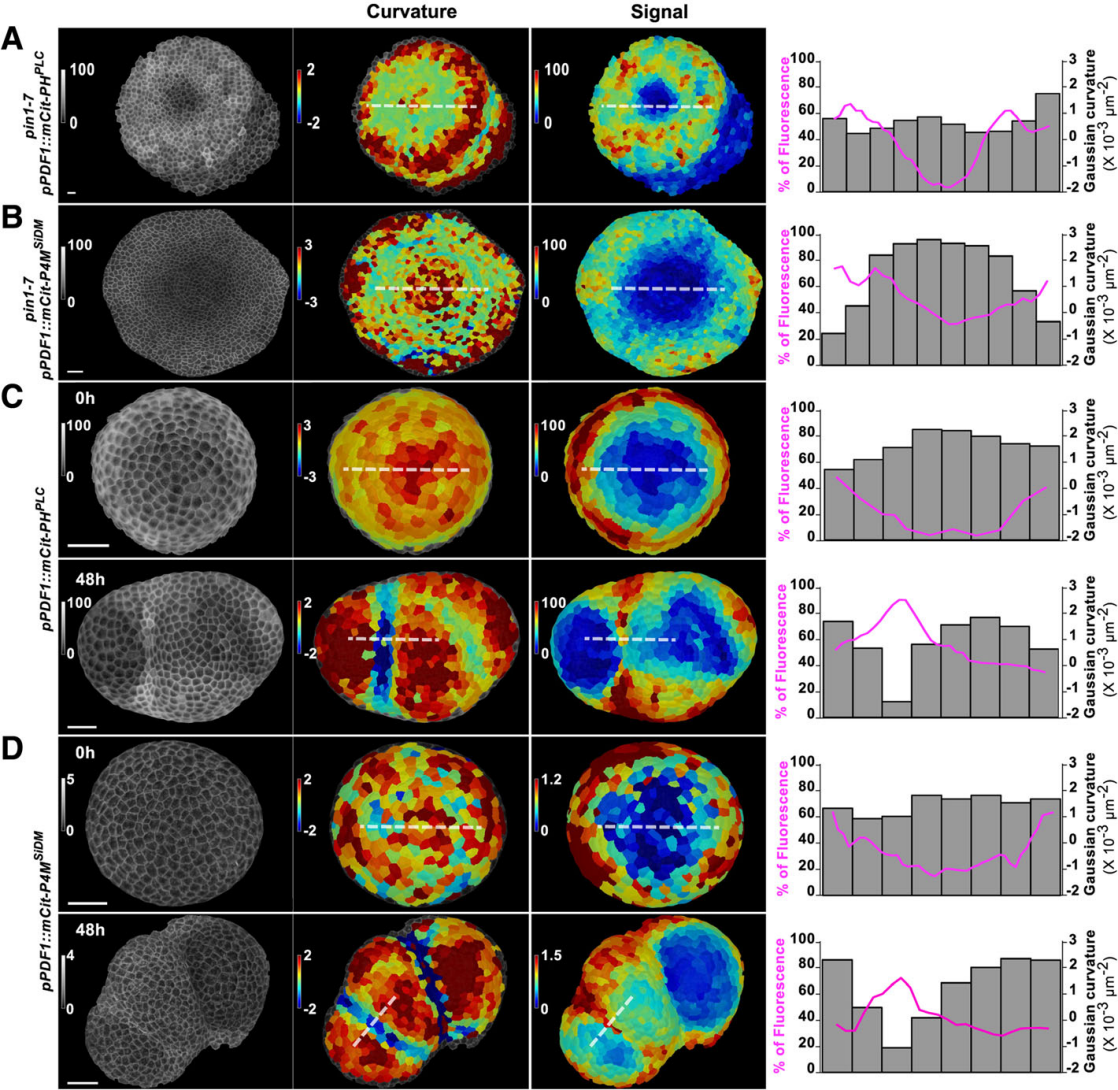
PIP pattern in absence of organogenesis at the shoot apical meristem. Epidermal signal intensity in shoot apical meristems (left panel, %) from (**a**, **b**) *pin1-7* mutant and (**c**, **d**) NPA-treated plants expressing (**a**, **c**) *pPDF1::mCitrine-PH*^*PLC*^ (**b**, **d**) and *pPDF1::mCitrine-P4M*^*SiDM*^. **c**, **d** NPA-treated seedlings were transferred to a medium without NPA at t = 0 h and observed at t = 48 h when organs emerged. Gaussian curvature (central panel, unit: 10^-3^ μm^-2^) and signal intensity (right panel, %) were extracted and plotted following the dotted white line. Scale bar, 20 μm

To check whether the PIP pattern is functionally associated with organogenesis, we introgressed the PIP biosensors in the *pin1-7* mutant [30], which is unable to generate flowers in inflorescences. In a *pin1-7* background, we observed minima of PI4P and PI(4,5)P_2_ signal intensity in the central zone, and maxima of PI4P and PI(4,5)P2 accumulation in the peripheral zone (Fig. 3a, b). When compared to the wild-type (WT), the PIP pattern appeared more homogeneous across the circumference of the peripheral zone, consistent with the absence of organ emergence in that background. Note also that, as in the WT, the differences between domains were more pronounced for the PI(4,5)P_2_ biosensor than the PI4P biosensor (Fig. 3a, b). These data suggest that the PIP pattern at the SAM can, at least in part, be uncoupled from organogenesis.

To further confirm this finding, we next modified auxin transport with 1-N-naphthylphthalamic acid (NPA). NPA-treated in vitro seedlings generate naked meristems, mimicking *pin1* meristems, arguably because NPA affects the recycling of PIN1 [31]. We observed qualitatively similar patterns in *pin1-7* mutant and in naked NPA-treated plants (Fig. 3c, d). Note that the pattern appeared more variable in NPA-treated plants than in *pin1-7* mutants, also correlating with variability in apex morphogenesis (e.g., Fig. 5a) and variability in auxin distribution in NPA-treated plants (Additional file 4: Figure S2). Interestingly, when taking NPA-treated plants off the drug, organs started to emerge at the periphery of the meristem, with the stereotypical PIP pattern observed in dissected meristems from greenhouse-grown plants (Fig. 3c, d).

Altogether, these data confirm the positive correlation between organogenesis and PI4P/PI(4,5)P_2_ accumulation in boundaries around emerging organs, and also suggest that the meristem maintains a pattern of PI4P and PI(4,5)P_2_ in the central and peripheral zones independent of organogenesis.

### Low PI4P and PI(4,5)P_**2**_ correlate with stem cell maintenance

**Fig. 4:**
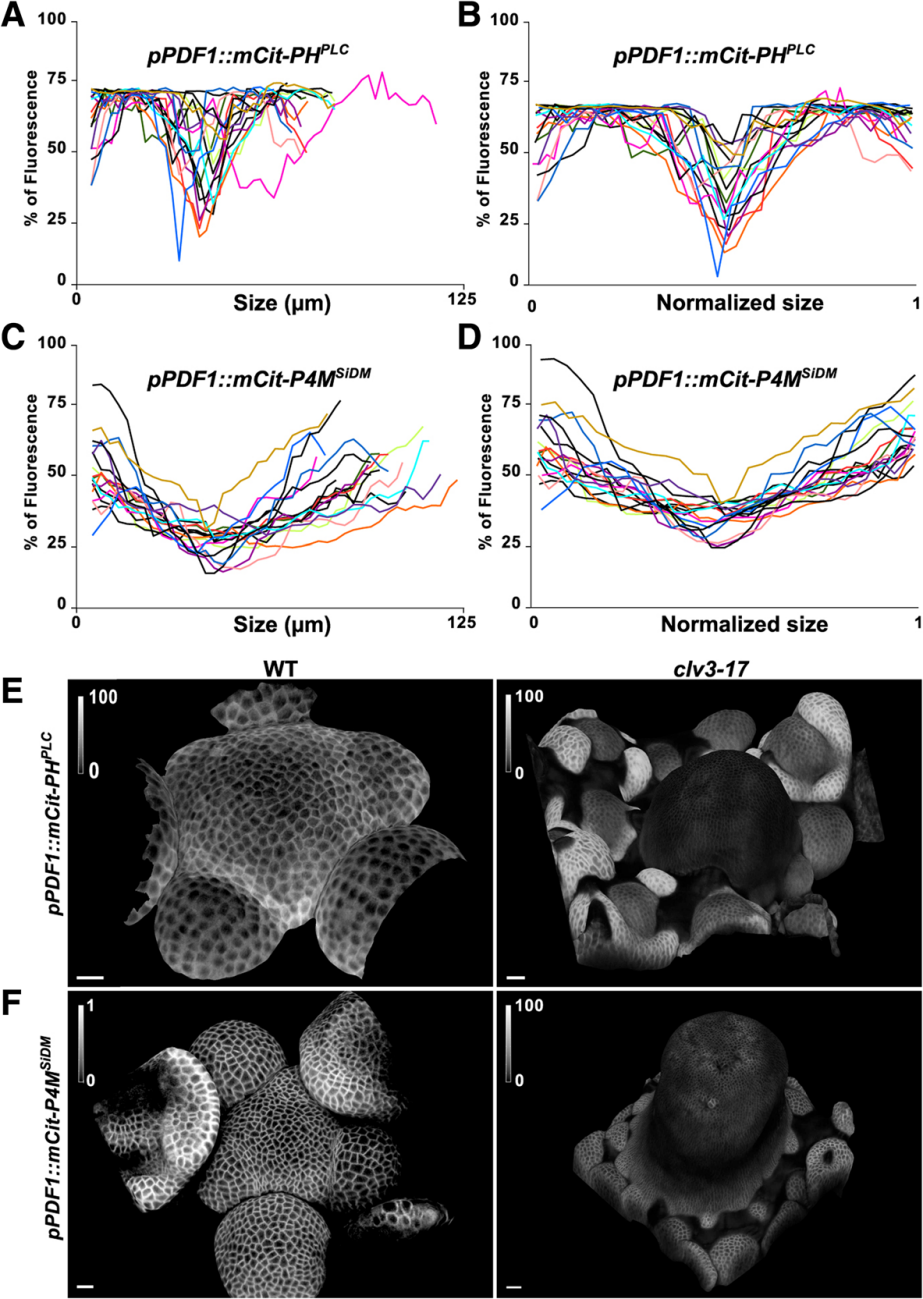
Low PI4P and PI(4,5)P2 levels correlate with stem cell maintenance. Signal intensity profile across the diameter of shoot meristems expressing (**a**, **b**) *pPDF1::mCitrine-PH*^*PLC*^ (n = 18) and (**c**, **d**) *pPDF1::mCitrine-P4M*^*SidM*^ (n = 18). (a, c absolute value and b, d relative values after normalization by the meristem diameter). The diameter of the *pPDF1::mCitrine-PH*^*PLC*^-depleted domain was equal to 20% of that of the whole meristem with a standard deviation of 0.05; the diameter of the *pPDF1::mCitrine-P4M*^*SidM*^-depleted domain was equal to 57% of that of the whole meristem, with a standard deviation of 0.144. Shoot meristems expressing (**e**) *pPDF1::mCitrine-PH*^*PLC*^ and (**f**) *pPDF1::mCitrine-P4M*^*SiDM*^ in Col-0 (left panel) or *clv3-17* (right panel). Note the presence of a few dead cells in the central zone of the meristem at the bottom right panel. Scale bar, 20 μm

Next, we investigated whether the reduction of PI4P and PI(4,5)P_2_ in the central zone correlates with stem cell maintenance. First, we took advantage of natural variability in meristem size to check the correlation between PIP depletion and central zone identity. In the WT, the central zone domain scales to meristem size – in larger meristems, cytokinin from the meristem epidermis is thought to diffuse over longer distances, leading to the activation of larger domains of WUS expression, which in turn would increase the population of CLV3-expressing cells in the central zone [32]. We reasoned that, if PIPs truly mark the central zone in the meristem, such scaling between meristem size and PIP depletion area in the central zone should be detected. Analyzing 18 meristems expressing PI4P biosensor and 18 meristems expressing PI(4,5)P_2_ biosensor, we found a range of meristem diameters from 81 to 122 μm (Fig. 4a, c). Strikingly, the PIP depleted zone in meristem scaled with meristem size, consistent with a scenario in which PIP accumulation negatively correlates with central zone identity (Fig. 4b, d).

To further check the relation between central zone identity and PIP accumulation, we analyzed the PI4P and PI(4,5)P_2_ accumulation in the *clv3-17* (SALK_065297) meristem, which exhibited a larger meristem, consistent with the de-repression of *WUS* in that background [3]. In *clv3-17* meristems, the PIP pattern largely disappeared (Fig. 4e, f). The signal was homogeneous and intensity was low, actually mimicking the PIP signature of the central zone in the WT. This demonstrates that the PIP pattern is under genetic control, and suggests that stem cell-ness is associated with reduced PIP accumulation and, potentially, with reduced PIP signaling. In principle, this may be sufficient to generate the observed PIP pattern at the SAM. Note that because PIP accumulation is low in the WT central zone and *clv3-17* meristems, the CLAVATA pathway is not likely to trigger PIP depletion in the WT central zone directly.

### PI(4,5)P_2_, cortical microtubules, and mechanical stress are positively correlated in the SAM

Many signals could contribute to the PIP pattern, and our results so far do not indicate the most simple scenario – depletion of PIP in the central zone could play a major role in the PIP pattern at the meristem, but is not sufficient to explain the accumulation of PIPs at the boundary; conversely, auxin is unlikely to be the main inducer of PIP accumulation since boundaries are largely auxin depleted and PIP concentration decreases in young primordia.

**Fig. 5:**
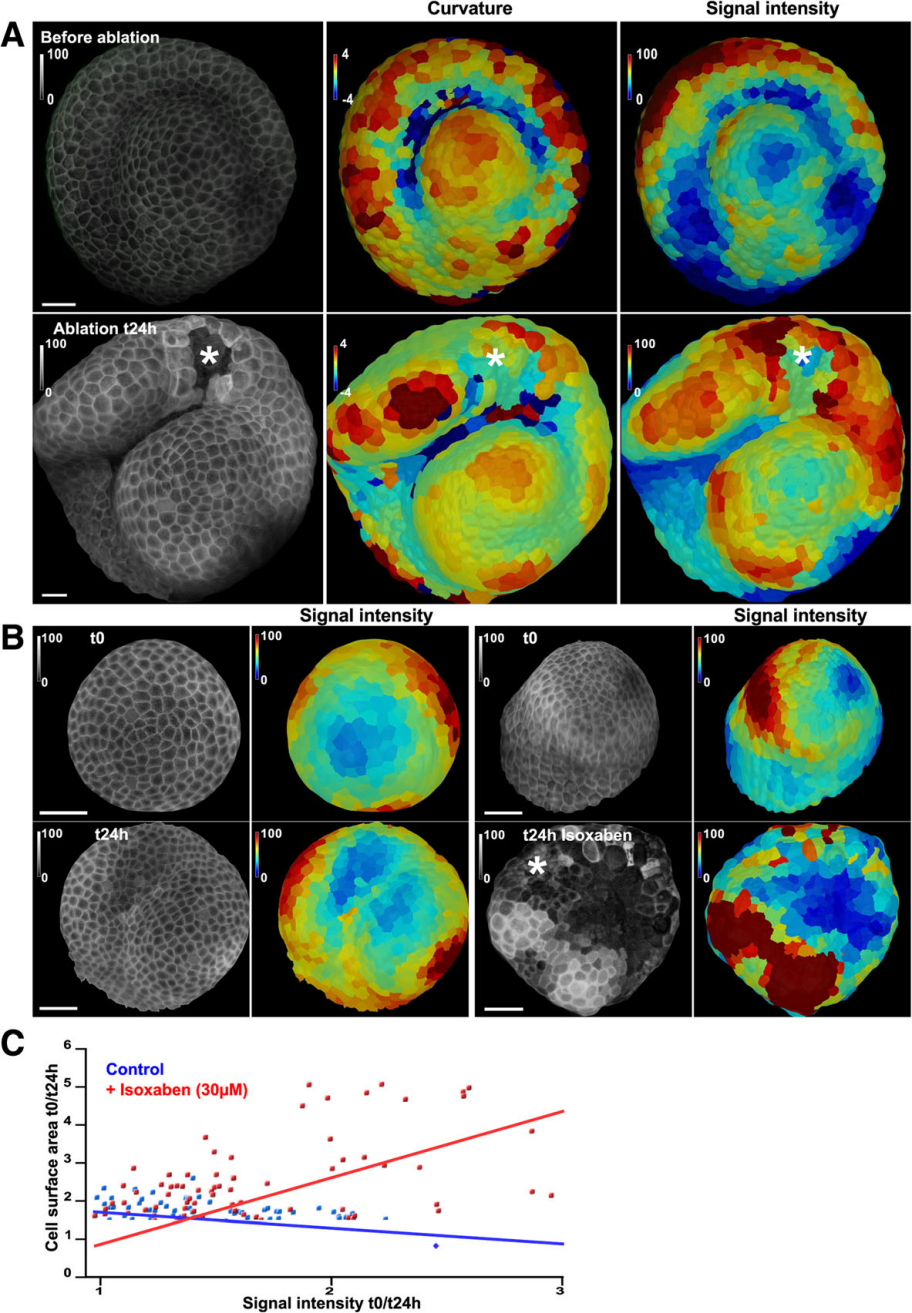
Induction of PIP2 after mechanical perturbations. **a** NPA-treated seedlings were transferred to a medium without NPA 1 day before t = 0 h. At t = 0 h, a small ablation was performed with a needle and the resulting impact on PIP2 biosensor signal intensity was recorded at t = 24 h. **b** NPA-treated seedlings were transferred to a medium without NPA 1 day before t = 0 h. At t = 0 h, whole plants were immersed in 30 μM isoxaben and the resulting impact on PIP2 biosensor signal intensity was recorded at t = 24 h. **c** Correlation between cell size and signal intensity with or without isoxaben treatment. Gaussian curvature (central panel, unit: 10^-3^ μm^-2^) and signal intensity (right panel, %) were extracted as in other figures. Scale bar, 20 μm

Interestingly, the *CUC3* and *STM* promoter activity at the SAM may provide the closest match to the observed PIP pattern. In particular, the accumulation of PIPs at the lateral sides of boundaries strongly recalls reported *CUC3* expression patterns [17, 18]. Interestingly, both promoters were recently shown to be activated by mechanical perturbations [17, 18]. To test whether mechanical stress contributes to the PIP pattern, we performed mechanical perturbations focusing on PI(4,5)P_2_, which exhibits the most clear-cut pattern. First, we performed local ablations with a small needle, knowing that such perturbations induce a circumferential tensile stress pattern (e.g., [11]) and observed a local PI(4,5)P_2_ accumulation near the ablated zone (Fig. 5a). Because ablations induce signals other than mechanical ones, we also modified the mechanical stress pattern using isoxaben, which inhibits cellulose synthesis and thus weakens cell walls and increases tensile stress levels (e.g., [33]). In these conditions, we observed an accumulation of PI(4,5)P_2_ (Fig. 5b). Strikingly, the cells that reached the largest size after isoxaben treatment, i.e., those that are likely experiencing higher stress, also exhibited higher biosensor signal intensity (Fig. 5c). Although we cannot rule out other, more complex, scenarios, these data strongly suggest that mechanical stress and PI(4,5)P_2_ accumulation are positively correlated.

**Fig. 6:**
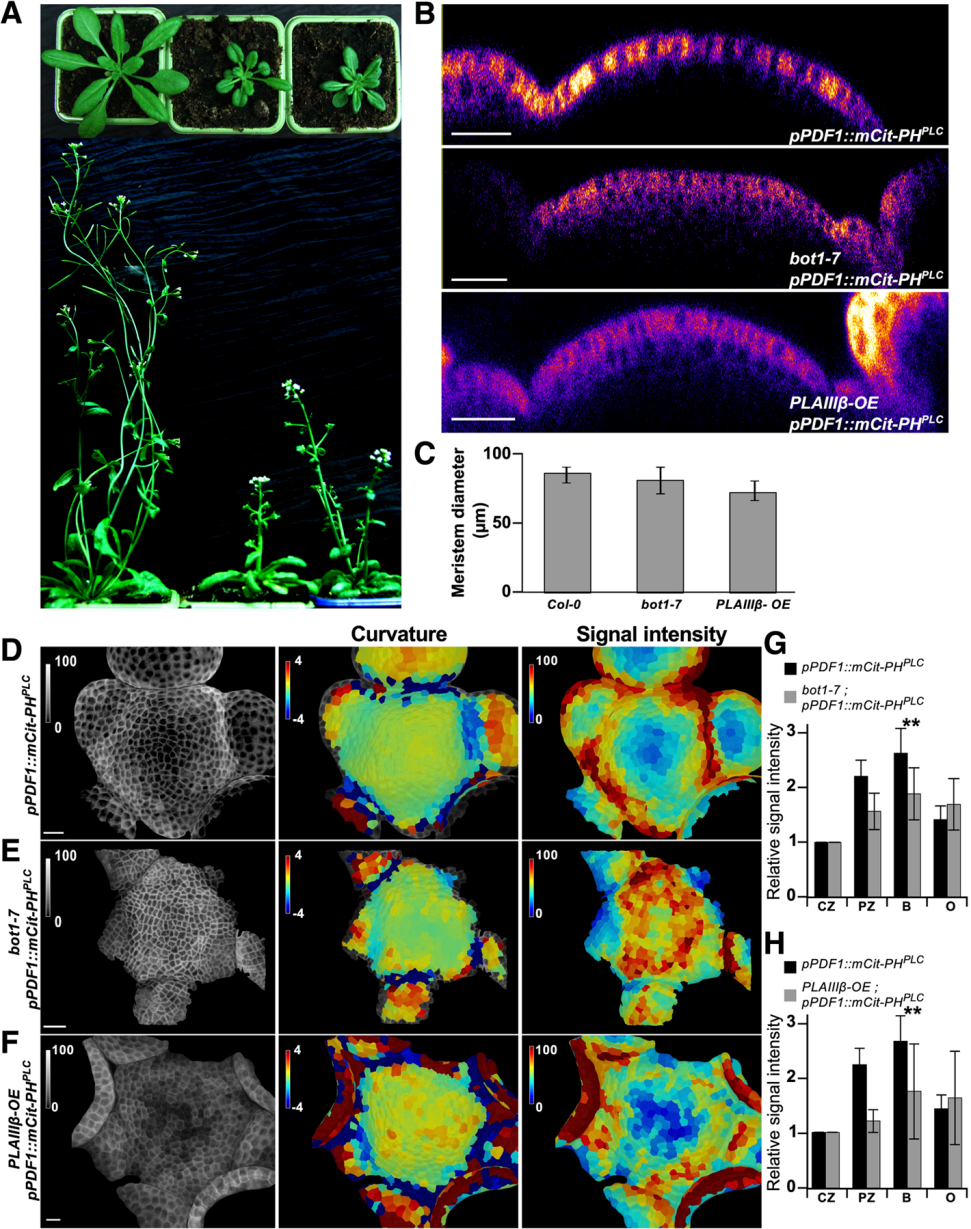
Flatter PIP pattern in the katanin mutant and *PLAIIIβ* overexpressor lines. **a** Whole plant phenotype in Col-0, *botero1-7* (*bot1-7*) and *phospholipase AIIIβ* overexpressor (*PLAIIIβ-OE*). **b** Transverse section through shoot apical meristems in Col-0, *bot1-7*, and *PLAIIIβ-OE* expressing *pPDF1::mCitrine-PH*^*PLC*^. **c** Meristem diameter in Col-0, *bot1-7*, and *PLAIIIβ-OE* expressing *pPDF1::mCitrine-PH*^*PLC*^. **d**–**f** Gaussian curvature (central panel, unit: 10^-3^ μm^-2^) and fluorescent signal intensity (left panel, %) in *pPDF1::mCitrine-PH*^*PLC*^ (**d**), *bot1-7 pPDF1::mCitrine-PH*^*PLC*^ (**e**), and *PLAIIIβ-OE pPDF1::mCitrine-PH*^*PLC*^ (**f**). (**g**–**h**) Signal intensity in the central zone (CZ) and boundaries (B) in *bot1-7* and *PLAIIIβ-OE* were compared to that of the wild-type. Statistical significance was tested using a bilateral Student test (**P < 0.01). Scale bar, 20 μm

Consistently, the PIP pattern also correlates with that of cortical microtubules, which align with a maximal tensile stress pattern. More specifically, we found that regions where cortical microtubules are more ordered (like boundaries and peripheral zones [11, 12, 33]) were also enriched in PIPs, whereas regions where cortical microtubule arrays have more isotropic orientations [34] were rather depleted in PIP (central zone and tip of emerging primordia). To further test the triple correlation between PIP accumulation, cortical microtubule order, and mechanical stress, we next analyzed PI(4,5)P_2_ pattern in *botero1-7*, a katanin mutant allele, which exhibits more disorganized microtubules, reduced growth anisotropy and slower microtubule response to mechanical perturbations because of its reduced severing activity and ability to self-organize [33, 35–37] (Fig. 6a). Although the global trends were maintained in that background, with lower PI(4,5)P_2_ concentration in the central zone and high PI(4,5)P_2_ concentration in the peripheral zone, the PI(4,5)P_2_ pattern in *bot1-7* was noisier than in the WT, and differences in PI(4,5)P_2_ levels between domains seemed less pronounced (Fig. 6d, e) – PI(4,5)P_2_ levels in boundaries were 1.81 higher than in the central zone in *bot1-7* (n = 3, *P* < 0.01) compared to a ratio of 2.63 (*P* < 0.001) in the WT.

The triple correlation between PI(4,5)P_2_, cortical microtubules, and mechanical stress at the SAM may also help us understand the role of PI(4,5)P_2_ in that tissue. To explore that question, we surveyed the literature for mutants and lines with potential links with mechanics, microtubule and phospholipids, and decided to revisit the phospholipase AIIIβ overexpressor phenotype. The exact relation between microtubules and phospholipids remains ill-described and, to date, there is no evidence of a functional link between mechanics, phospholipids, and microtubules at the SAM. The published phospholipase AIIIβ overexpressor phenotype largely echoes that of the katanin mutant, with dwarfism and isotropic growth; the presence of brittle stems in both backgrounds also suggests a relation with mechanics [38, 39]. In contrast to phospholipase C and D, which respectively cleave phospholipids before and after the phosphate, and respectively release diacylglycerol and phosphatidic acid in the membrane, phospholipase A cleaves after the last carbon of the fatty acid chain and releases a lysophospholipid and a free fatty acid. We generated new phospholipase AIIIβ overexpressor lines and confirmed, in our growth conditions, that the *bot1-7* mutant and phospholipase AIIIβ overexpressor exhibit comparable plant phenotypes (Fig. 6a). Meristem shapes were also affected in both backgrounds, where the *bot1-7* mutant exhibited a flatter meristem (as shown previously [33]) and the phospholipase AIIIβ overexpressor meristems were significantly smaller than that of the WT (Fig. 6c). Although the PI(4,5)P_2_ depleted central zone was more clearly visible in the phospholipase AIIIβ overexpressor than in *bot1-7* (Fig. 6d–f), the gradient in PI(4,5)P_2_ accumulation was less pronounced in both backgrounds when compared to the WT (Fig. 6d–f). The PI(4,5)P_2_ pattern was also more noisy in both backgrounds when compared to the WT, as shown by high standard deviations in peripheral zones and boundaries (Fig. 6g, h).

The striking phenotypic similarities between *bot1-7* and phospholipase AIIIβ overexpressor, together with the defective PI(4,5)P_2_ patterns in both genetic contexts thus suggests that the nexus between microtubule and mechanical stress at the SAM depends on the phospholipid pattern.

## Discussion

**Fig. 7:**
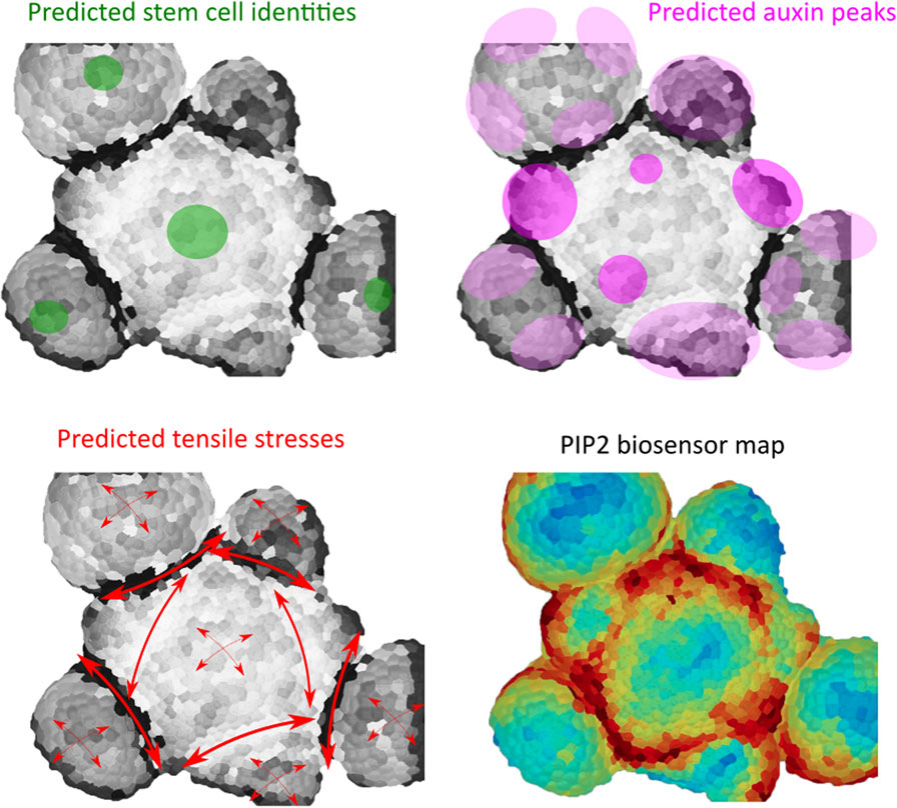
Graphical abstract. Predicted patterns of stem cell identities, auxin peaks and tensile stress are represented on the curvature map of a meristem that expresses the PI(4,5)P_2_ biosensor, revealing negative correlations between PI(4,5)P_2_ accumulation and stem cell identity and a positive correlation between PI(4,5)P_2_ accumulation and anisotropic tensile stresses

Altogether, we show the existence of a stereotypical PIP pattern at the meristem, with PI(4,5)P_2_ exhibiting a more clear-cut pattern than PI4P, and where PIPs accumulate in boundaries and are depleted in the central zone. Our analysis in *pin1*, *clv3*, and *katanin* mutants and phospholipase AIIIβ overexpressor suggest that this pattern is the result of multiple signals, and reveals a triple positive correlation between mechanical stress, cortical microtubules, and PI(4,5)P_2_ accumulation (Fig. 7).

Reports relating phospholipids with meristem functions are rare. Two protein phosphatase type 2C proteins, POLTERGEIST (POL) and POL-like 1 (PLL1), have been involved in stem cell identity through a WUS induction activity [40, 41]. Both proteins are acylated and their localization to plasma membrane is required for their function – while PLL1 overexpression leads to over-accumulation of stem cells in inflorescence meristems, overexpression of a PLL1 with mutation in acylation domains, and thus unable to bind membranes, does not affect stem cell number [41]. POL and PLL1 can directly interact with PI4P [42], yet the exact relation between POL, PLL1, and plasma membrane composition remains to be investigated. More directly related to anionic phospholipids, the phosphatidylserine synthase mutant *pss1* exhibits growth delays, sterile flowers, smaller meristems, a reduced division rate, reduced *CLV3* and *WUS* expression, and increased expression of *KNOX* genes like *STM* or *BP* [43]. In the simplest scenario, this would suggest that phosphatidylserine promotes *CLV3* and *WUS* expression, and hinders *KNOX* expression. Interestingly, our data suggest that PI(4,5)P_2_, and to a smaller extent PI4P, levels are high in boundaries (where *KNOX* expression is also high) and are low in the central zone (where *CLV3* expression is high), i.e., opposite to the predicted role of phosphatidylserine. Although this shows that the exact relation between phosphoinositides and the expression of master meristem regulators is not straightforward, these data suggest that phosphoinositides are important contributors to meristem functions.

Focusing on organogenesis, the PIP pattern may in fact better match structural aspects of the meristem. The katanin mutant exhibits major shape defects, with a crater morphology and shallow boundaries [33]. Here, we show that the PIP distribution is also flatter in that background, further strengthening the relation between PIP pattern and mechanical features (shape and growth reflecting cell and tissue mechanics). The katanin mutant also exhibits decreased growth heterogeneity and increased cell size heterogeneity in the SAM [33]; here, we find increased heterogeneity in PIP levels within the peripheral zone of the katanin mutant meristems, which may also be consistent with the identified correlation between cell size, mechanical stress, and PIP2 accumulation after isoxaben treatment in the present study. Conversely, the katanin mutant also displays WT features, namely the *CLV3* expression domain is not majorly affected [32, 33], organogenesis occurs roughly at the same rate, and phyllotaxis (spatial position of consecutive organs in the SAM) appears normal [44]. The present data may thus help us distinguish between PIP-dependent and PIP-independent functions at the SAM.

To explain the correlation between PIP and mechanical features, one could involve a direct interaction between PIPs and cortical microtubules. Analysis of cortical microtubules with high-resolution scanning electron microscopy in *Tradescantia virginiana* revealed a physical linkage between microtubules and the plasma membrane [45]. Phospholipase D δ isoform (PLDδ) is able to bind microtubules as well as the plasma membrane [19, 46], and its activation triggers microtubule reorganization [47]. This anchoring may also require the PLD-signaling product, phosphatidic acid (PA), where PA specifically recruits the microtubule-associated protein 65-1 (MAP65-1) [48]. Consistently, in vitro analysis showed that β-tubulin can be pulled down with GFP-PLDδ from Arabidopsis suspension cells [49]. Yet, reports of putative interactions between PIPs and microtubules remain scarce.

In an alternative scenario, mechanical signals could play a role in patterning PIPs, through other cytoskeletal elements or signaling factors. There is increasing evidence for a role of PIPs in cytoskeletal dynamics and response to mechanical forces in animal systems [50]. Notably, using optical tweezers, it was found that PIP_2_ regulates the adhesion energy between the actin cytoskeleton and plasma membrane [20]. Computational modeling also suggests that PIP_2_ may alter the conformation of mechanosensors like integrin [21]. Similarly, TRP mechanosensitive channels are activated upon PIP_2_ hydrolysis [22, 51]. This opens exciting avenues for comparative analysis with plant cells.

## Conclusions

By uncovering the pattern of PIPs at the SAM, we attempted to integrate the role of PIPs in meristem biology and found a negative correlation with stem cell maintenance on the one hand, and a positive correlation with morphogenesis and mechanical stress on the other (Fig. 7). As PIPs are likely involved in many signaling pathways, their exact position in the meristem gene network will certainly help us understand the nexus between structure and function at the meristem. Conversely, as the meristem appears as a good multicellular system to study the role of mechanical signals, the analysis of PIPs at the meristem may provide an interesting point of comparison with PIP function in mechanosensing in animals.

## Methods

### Plant growth conditions

Seeds were surface sterilized, stratified for 3 days at 4 °C in the dark, and sown on Arabidopsis medium (Duchefa, Haarlem, the Netherlands). Plants were then transferred on soil, first in short-day conditions (8 h/16 h light/dark period and 70% humidity) for 1 month and then transferred to long-day conditions (16 h/8 h light/dark period and 70% humidity).

### Mutants and transgenic lines

We used the following lines: Columbia 0 (Col-0), *pin1-7* [30], *bot1-7* [33], *clv3-17* (SALK_065297), DII-VENUS [15], and *pUbQ10::29-1-TdTomato* [52]. We used the following primers to genotype the *clv3-17* plants: 5' ATG ACA TTG GAG GAA CGA AAG 3', 5' TGT AGA TGT CCG GTC CAG TTC 3', and LBB1 (SALK primer). Domains PH^PLC^ and P4M^SiDM^ fused to the fluorophore mCitrine have been previously described in [23] and [24], respectively. To generate the *PDF1::mCitrine-PH*^*PLC*^, *PDF1::mCitrine-P4M*^*SiDM*^, *pPDF1::29-1-TdTomato*, and *p35S::PLAIIIβ* plasmids, the MultiSite Gateway Three-Fragment strategy (Life Technologies) was used; namely, a 1456 pb promoter sequence upstream of *PDF1* [17], the Cauliflower mosaic virus promoter (*CaMV35S*), the *29-1-TdTomato* sequence (1764 pb) amplified from plant expressing *pUbQ10::29-1-TdTomato* [52], the fluorophore mCitrine [53], the PH^PLC^ or P4M^SiDM^ domains [23, 24], and coding sequences of PLAIIIβ (AT3G54950) were subcloned into multiple Gateway cassettes with flanking attB sites. Corresponding Gateway cassettes were inserted in pBART (basta resistant) for *pPDF1::mCitrine-PH*^*PLC*^, *pPDF1::mCitrine-P4M*^*SiDM*^, and *p35S::PLAIIIβ* or pART (kana resistant) for *pPDF1::29-1-TdTomato* as destination vector. Col-0 plants were transformed by dipping inflorescences as described previously [23]. For the generation of these transgenic lines, we followed the national guidelines and legislations, having the license to do so (GMO charter).

### Confocal laser scanning microscopy and sample preparation

Shoot apices were dissected as previously described [27]. To generate naked meristems in vitro, seeds were directly sown on Arabidopsis medium supplemented with 10 μM NPA. Seedlings with naked meristems were then transferred to a medium without NPA as described before [31]. Confocal imaging was performed on a Leica SP8 upright scanning confocal microscope equipped with a water immersion objective (HCX IRAPO L 25x/0.95 W). Fluorophores were excited using Led laser (Leica Microsystems, Wetzlar, Germany) emitting at wavelengths of 448 nm for CFP, 514 nm for mCitrine, and 552 nm for TdTomato and FM4-64. Images were collected at 464–500 nm for CFP, 521–550 nm for mCitrine, 610–650 nm for TdTomato, and 650–700 nm for FM4-64. The following scanning settings were used: pinhole size 1AE, 1.25x zoom, scanning speed of 8000 Hz (resonant scanner), frame averaging 4, Z intervals of 0.5 μm. Membrane staining was performed by adding a drop of FM4-64 (0.1 mg/mL) directly on top of the dissected meristem.

### Ablations and isoxaben treatments

Ablations and isoxaben treatments that were carried out on WT plants were performed on plants previously grown in vitro in NPA and transferred into a medium without NPA 0–24 hours before the beginning of the experiment. The ablations were performed with a needle. The isoxaben treatments were conducted by immersing plants in an aqueous solution of isoxaben (30 μM). Controls were obtained by water immersion with an equivalent volume of dimethyl sulfoxide. Controls and assays were analyzed in parallel (same growth conditions and imaging conditions).

### Image analysis

The maps and quantifications of meristem curvature and signal intensity at cellular levels were obtained using the MorphographX (MGX) software (http://www.mpipz.mpg.de/MorphoGraphX/ [25]) according to a previously described procedure [27]. The curvature maps displayed in the figures correspond to Gaussian curvatures with a neighboring of 15 μm. The signal intensity maps were generated by extracting the fluorescent signal at the surface of the meristem (up to 2 μm from the meristem surface) and by projecting it on the cellular mesh. Quantification of signal intensity in the central zone, peripheral zone, boundary and organs were done by seeding the four domains in MGX and extracting the corresponding signal intensity. Extraction of meristem curvature and signal intensity along a transect was done using ImageJ (https://sites.google.com/site/qingzongtseng/template-matching-ij-plugin) by drawing a 10-μm thick line between a young organ and the central zone. Note that fluorescent signal intensity is represented as a percentage of maximal intensity (saturation). We set similar laser intensity in WT and mutants as follows: 10% for *pPDF1::mCitrine-PH*^*PLC*^, 1% for *pPDF1::mCitrine-P4M*^*SiDM*^, 1% for *pPDF1::29-1-TdTomato*, and 5% for *pUbQ10::29-1-TdTomato* and FM4-64 stained meristems. Sample size and *t* tests are presented in Additional file 2: Table S1 and raw data in Additional file 3: Table S2.

### Statistical analysis

A non-parametric Mann–Whitney *U* test was applied to test differences in signal intensity between the central zone and the boundary, and to compare SAM size in the different genotypes.

## Additional files

Additional file 1: Figure S1. Distinction between zones at the shoot apex, based on curvature. From the curvature map, the boundary (B) could be recognized by its negative Gaussian curvature (light green to blue). Organs (O) are located outside of the boundaries and exhibit highly positive Gaussian curvature (orange to red). Old organs were excluded from the analysis. The meristem was subdivided into central zone (CZ) and peripheral zone (P), assuming that the thickness of peripheral zone ring is roughly similar to the diameter of the central zone. (PNG 167 kb)

Additional file 2: Table S1. Sample size and statistical analysis. (ODS 5 kb)

Additional file 3: Table S2. Raw data (relative signal intensities) for individual apices. (ODS 5 kb)

Additional file 4: Figure S2. Variable auxin pattern in NPA-treated plants expressing DII-Venus. Shoot apical meristems from seedlings grown on NPA-containing medium from germination. At t = 0 h, plants were taken off the drug. Scale bar, 20 μm. (JPG 1268 kb)
