# Supplementary figures and images for: A phosphoinositide map at the shoot apical meristem in Arabidopsis thaliana
Source: BMC Biol. 2018 Feb 7;16:20. doi: 10.1186/s12915-018-0490-y (PMC5803925; doi:10.1186/s12915-018-0490-y)

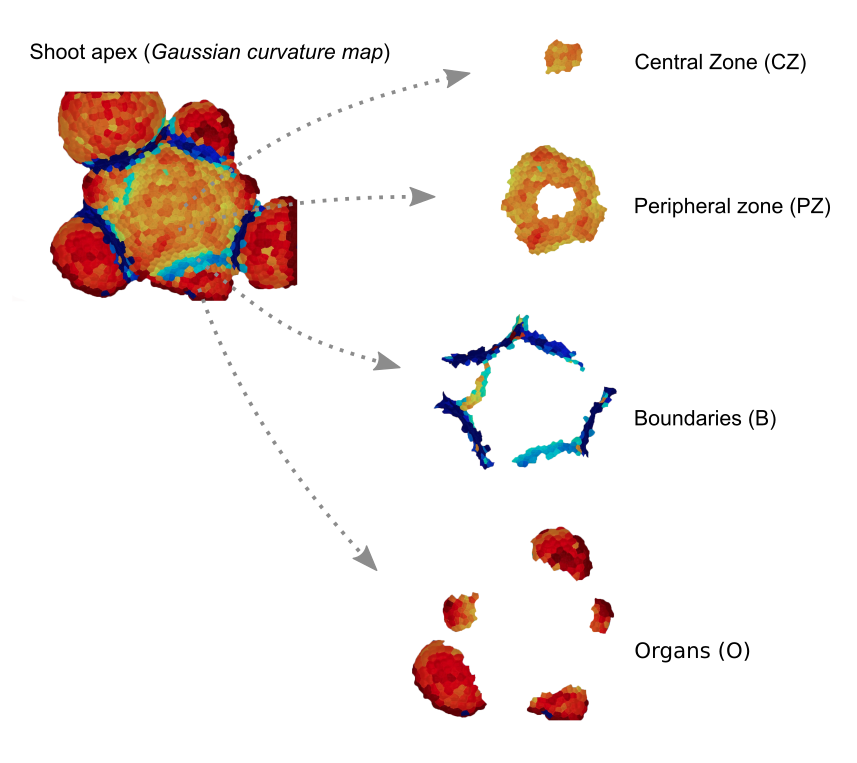

Supplement: Supplementary file 1 — Distinction between zones at the shoot apex, based on curvature. From the curvature map, the boundary (B) could be recognized by its negative Gaussian curvature (light green to blue). Organs (O) are located outside of the boundaries and exhibit highly positive Gaussian curvature (orange to red). Old organs were excluded from the analysis. The meristem was subdivided into central zone (CZ) and peripheral zone (P), assuming that the thickness of peripheral zone ring is roughly similar to the diameter of the central zone. (PNG 167 kb) [file 12915_2018_490_MOESM1_ESM.png]

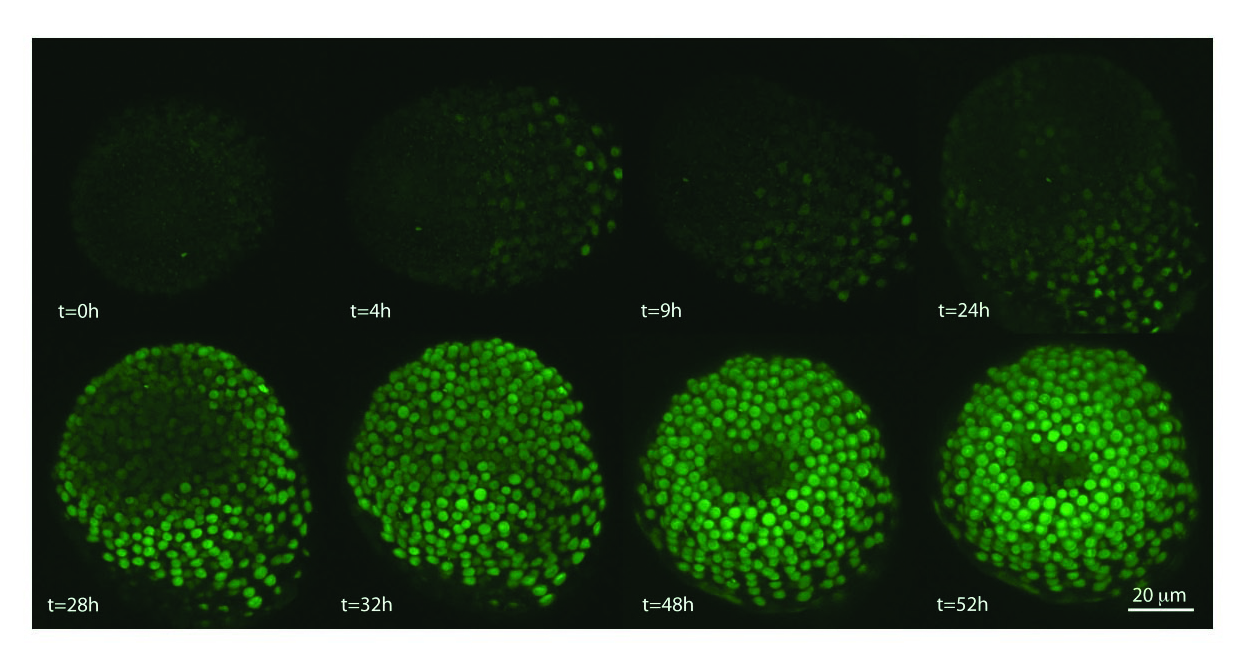

Supplement: Supplementary file 4 — Variable auxin pattern in NPA-treated plants expressing DII-Venus. Shoot apical meristems from seedlings grown on NPA-containing medium from germination. At t = 0 h, plants were taken off the drug. Scale bar, 20 μm. (JPG 1268 kb) [file 12915_2018_490_MOESM4_ESM.jpg]
